# ﻿A further study on the spider genus *Baiyuerius* Zhao, Li & Li, 2023, from China (Agelenidae, Coelotinae)

**DOI:** 10.3897/zookeys.1184.107931

**Published:** 2023-11-15

**Authors:** Bin Luo, Feng Lu, Zhi-Sheng Zhang, Lu-Yu Wang

**Affiliations:** 1 Key Laboratory of Eco-environments in Three Gorges Reservoir Region (Ministry of Education), School of Life Science, Southwest University, Chongqing 400715, China Southwest University Chongqing China; 2 College of Life Sciences and Oceanography, Shenzhen University, Shenzhen 518000, China Shenzhen University Shenzhen China

**Keywords:** Distribution of new species, morphology, new combination, redescription, taxonomy

## Abstract

*Baiyuerius* is a newly erected genus of Coelotinae spiders comprising five species distributed in southern China and northern Vietnam. Two additional new species, *B.shenzhen***sp. nov.** (male and female) and *B.yuelu***sp. nov.** (male and female), are described here. Three new combinations are proposed, namely *Baiyueriusacroprocessus* (Zhang, Zhu & Wang, 2017) **comb. nov.**, *Baiyueriusglobasus* (Wang, Peng & Kim, 1996) **comb. nov.** and *Baiyueriusrugosus* (Wang, Peng & Kim, 1996) **comb. nov.** Descriptions, photographs, and a distribution map of the known and newly proposed species are provided.

## ﻿Introduction

The subfamily Coelotinae comprises about 806 species in 40 genera distributed across the northern hemisphere. It is particularly abundant in east Asia (WSC 2023), and nine new genera from China have been erected in recent years (Li et al. 2018): *Baiyuerius* Zhao, B. Li & S.Q. Li, 2023; *Guilotes* Zhao & S.Q. Li, 2018; *Hengconarius* Zhao & S.Q. Li, 2018; *Jishiyu* Lin & Li, 2023; *Nuconarius* Zhao & S.Q. Li, 2018; *Sinodraconarius* Zhao & S.Q. Li, 2018; *Troglocoelotes* Zhao & S.Q. Li, 2019; *Vappolotes* Zhao & S.Q. Li, 2019; *Yunguirius* B. Li, Zhao & S.Q. Li, 2023. The genus *Baiyuerius* includes five species from southern China and northern Vietnam.

While examining specimens of Coelotinae spiders, two new species were discovered from Hunan and Guangdong provinces: *Baiyueriusyuelu* sp. nov. and *B.shenzhen* sp. nov. The type specimens of *Draconariusacroprocessus* Zhang, Zhu & Wang, 2017, *Coelotesglobasus* (Wang, Peng & Kim, 1996) and *Corasrugosus* (Wang, Peng & Kim, 1996) were examined, and these species are transferred to the genus *Baiyuerius*.

## ﻿Materials and methods

All specimens are preserved in 75% ethanol and were examined, illustrated, photographed, and measured using a Leica M205A stereomicroscope, a Leica DFC450 Camera, and LAS software (v. 4.6). Male pedipalps and epigynes were examined and illustrated after dissection. Epigynes were cleared in pancreatin (Álvarez-Padilla and Hormiga 2007). Leg measurements are shown as total lengths (coxa + trochanter, femur, patella, tibia, metatarsus, and tarsus). All measurements are in millimeters.

Morphological terminology follows Zhao et al. (2023). The following abbreviations are used in the text and figure legends: A, atrium; ALE, anterior lateral eye; AME, anterior median eye; C, conductor; CD, copulatory duct; CDA, dorsal apophysis of conductor; CF, cymbial furrow; CO, copulatory opening; E, embolus; EB, embolic base; FD, fertilization duct; H, hood; LTA, lateral tibial apophysis; MA, median apophysis; PA, patellar apophysis; PES, posterior epigynal sclerite; PLE, posterior lateral eye; PME, posterior median eye; MOA, median ocular area; RTA, retrolateral tibial apophysis; S, spermatheca; SE, swell of epigyne; ST, subtegulum; T, tegulum.

All specimens examined here are deposited in the School of Life Sciences, Southwest University, Chongqing, China (**SWUC**) and College of Life Sciences, Hunan Normal University, Changsha, China (**HNU**).

## ﻿Results

### ﻿Taxonomy

**Family Agelenidae C.L. Koch, 1837** (漏斗蛛科)

**Subfamily Coelotinae F.O. Pickard-Cambridge, 1893** (隙蛛亚科)

**Genus *Baiyuerius* Zhao, Li & Li, 2023** (百越蛛属)

With the addition of five more species to *Baiyuerius*, the diagnostic characters of the genus are revised here. Base of the male pedipalp cymbium enlarged, with one or two hypophyses; conductor long membranous, beak-shaped, ventrally grooved. Females identified by position of copulatory opening at center of atrium mid-ventrally; copulatory ducts form long loop extending anteriorly before entering spermathecae; atrium located anteriorly and occupies less than or equal to half of epigyne; spermatheca simple to highly convoluted (Zhao et al. 2023).

#### ﻿ Baiyuerius acroprocessus

Taxon classificationAnimaliaAraneaeAgelenidae

(Zhang, Zhu & Wang, 2017) comb. nov.

682598C8-F255-50FC-B43D-94F00EF261A7

Figs 1 , 7 (顶突百越蛛)

Draconarius acroprocessus Zhang, Zhu & Wang in Zhu et al. 2017: 220, fig. 107A–C (♂).

##### Material examined.

1 male (holotype, SWUC-T-AG-19-01): China, Hubei Province, Xuanen County, Jiaoyuan Town, 30 Augustus 2004, Z.S. Zhang & H.M. Chen leg.

##### Diagnosis.

**Figure 1.**
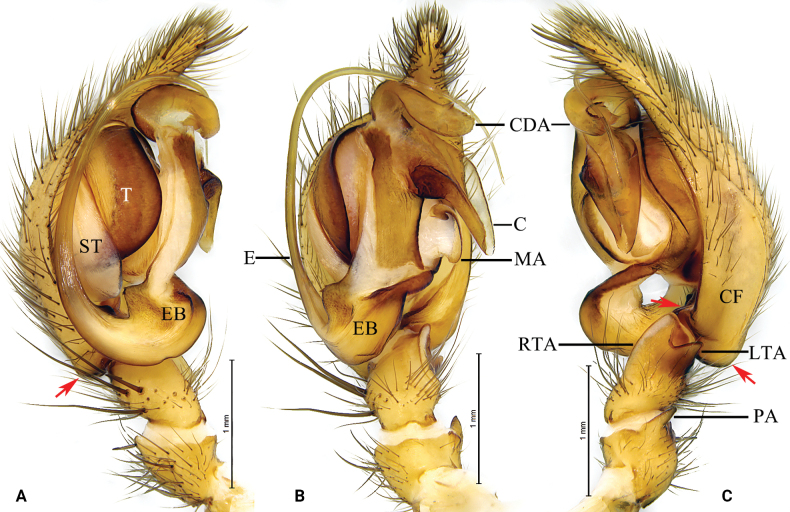
*Baiyueriusacroprocessus* (Zhang, Zhu & Wang, 2017), comb. nov., male holotype **A** left male pedipalp, prolateral view **B** same, ventral view **C** same, retrolateral view. Arrows show the hypophysis of cymbium. Abbreviations: A = atrium; C = conductor; CDA = dorsal apophysis of conductor; CF = cymbial furrow; E = embolus; EB = embolic base; LTA = lateral tibial apophysis; MA = median apophysis; PA = patellar apophysis; PES = posterior epigynal sclerite; RTA = retrolateral tibial apophysis; ST = subtegulum; T = tegulum.

Male resembles that of *B.zhuping* Zhao, Li & Li, 2023 in having the similar patellar apophysis with a pointed distal end, cymbial furrow almost half the length of cymbium (Fig. 1; Zhao et al. 2023: fig. 5A–C), but *B.acroprocessus* comb. nov. can be distinguished from the latter by the following: conductor dorsal apophysis with bifurcated apex and strongly sclerotized (Fig. 1A–C) vs unbifurcated in *B.zhuping* (Zhao et al. 2023: fig. 5A–C); embolic base margin serrated in retrolateral view (Fig. 1A–C) vs embolic base smooth in *B.zhuping* (Zhao et al. 2023: fig. 5A–C); cymbial base with two hypophyses (Fig. 1A–C) vs with one hypophysis in *B.zhuping* (Zhao et al. 2023: fig. 5A–C).

##### Description (partial).

Pedipalp (Fig. 1A–C): patellar apophysis thumb-shaped with pointed end; retrolateral tibial apophysis originating from midway along length of tibia; lateral tibial apophysis somewhat triangular, with blunt end; cymbial furrow half as long as cymbium; cymbial base with two hypophyses; median apophysis spoon-like; embolus originating at 7 o’clock; conductor translucent, with smooth, wrinkled surface and serrated margin; conductor dorsal apophysis with bifurcated apex and strongly sclerotized. Habitus as shown by Zhu et al. (2017). Female unknown.

##### Distribution.

China (Hubei) (Fig. 7).

#### ﻿ Baiyuerius globasus

Taxon classificationAnimaliaAraneaeAgelenidae

(Wang, Peng & Kim, 1996) comb. nov.

55034C19-C9D8-564A-A795-A11DC7AF9B15

Figs 2 , 7 (球百越蛛)

Coras globasus Wang et al. 1996: 77, figs 1–3 (♀); Song et al. 1999: 388, fig. 229C, D (♀); Yin et al. 2012: 998, fig. 512a, b (♀).
Coelotes globasus : Wang and Jäger 2007: 28, figs 16–18; Zhu et al. 2017: 164, fig. 73A, B (♀).

##### Material examined.

1 female (holotype, HNU): China, Hunan Province, Zhangjiajie, 17 October 1984, J.F. Wang leg.

##### Diagnosis.

**Figure 2.**
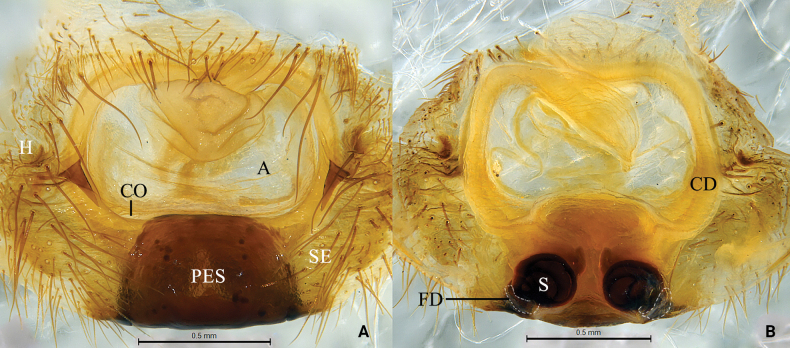
*Baiyueriusglobasus* (Wang, Peng & Kim, 1996), comb. nov., female holotype **A** epigyne, ventral view **B** same, dorsal view (dash-line indicating the outline of fertilization ducts). Abbreviations: A = atrium; CD = copulatory duct; CO = copulatory opening; FD = fertilization duct; H = hood; PES = posterior epigynal sclerite; S = spermatheca; SE = swell of epigyne.

Female resembles that of *B.zuojiang* Zhao, Li & Li, 2023 in having the similarly round spermathecae (Fig. 2; Zhao et al. 2023: fig. 7A–C), but can be distinguished from the latter by the following: atrium somewhat rectangular in *B.globasus* comb. nov. (Fig. 2A) vs glasses-shaped in *B.zuojiang* (Zhao et al. 2023: fig. 7A); epigyne with conspicuous epigynal teeth in *B.globasus* comb. nov. (Fig. 2A) vs epigynal teeth absent in *B.zuojiang* (Zhao et al. 2023: fig. 7A); copulatory opening present mid-ventrally in the atrium in *B.globasus* comb. nov. (Fig. 2B) vs present in the anterio-lateral margin of the atrium in *B.zuojiang* (Zhao et al. 2023: fig. 7A); copulatory ducts strongly curved; anterior end touches each other in *B.globasus* comb. nov. (Fig. 2B) vs arc-shaped, anterior end present laterally apart away from each other in *B.zuojiang* (Zhao et al. 2023: fig. 7B).

##### Description (partial).

Epigyne (Fig. 2A, B): atrium somewhat rectangular; epigynal hoods present laterally; epigynal teeth distinct; copulatory opening located mid-centrally; copulatory ducts originating centrally, extending anteriorly, then curved inward, anterior ends touches each other; spermathecae fist-shaped; fertilization ducts transparent. Habitus as shown by Wang et al. (1996)

Male unknown.

##### Distribution.

China (Hunan) (Fig. 7).

#### ﻿ Baiyuerius rugosus

Taxon classificationAnimaliaAraneaeAgelenidae

(Wang, Peng & Kim, 1996) comb. nov.

0B3C80F8-12EC-51D6-94CE-19F3819BE5D2

Figs 3 , 7 (皱纹百越蛛)

Coras rugosus Wang et al. 1996: 78, figs 4–6 (♀); Song et al. 1999: 388, fig. 229E, F (♀); Yin et al. 2012: 999, fig. 513a–c (♀).
Coelotes rugosus : Wang and Jäger 2007: 33, figs 41–43; Wang and Jäger 2010: 1176, fig. 2H (♀); Zhu et al. 2017: 186, fig. 94A, B (♀).

##### Material examined.

1 female (holotype, HNU): China, Hunan Province, Chenbu County, 1982, Y. Liu leg.

##### Diagnosis.

**Figure 3.**
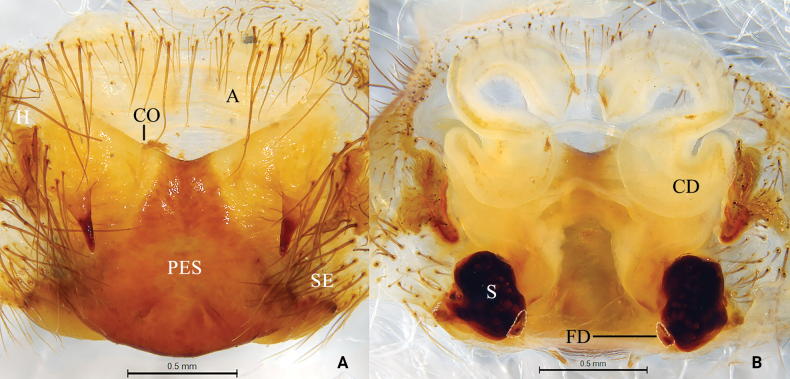
*Baiyueriusrugosus* (Wang, Peng & Kim, 1996), comb. nov., female holotype **A** epigyne, ventral view **B** same, dorsal view (dash-line indicating the outline of fertilization ducts). Abbreviations: A = atrium; CD = copulatory duct; CO = copulatory opening; FD = fertilization duct; H = hood; PES = posterior epigynal sclerite; S = spermatheca; SE = swell of epigyne.

Female resembles *B.yuelu* sp. nov. in having atrium similar and conspicuous epigynal teeth (Fig. 6; Zhao et al. 2023: fig. 7A–C) but distinguished by the following: spermathecae highly convoluted (Fig. 3B) vs round in *B.yuelu* sp. nov. (Fig. 6C); copulatory ducts with two turns (Fig. 3B) vs with one turn in *B.yuelu* sp. nov. (Fig. 6C).

##### Description (partial).

Epigyne (Fig. 3A, B): atrium glasses-shaped, occupying 1/3 of epigyne; epigynal hood located laterally; epigynal teeth distinct; copulatory opening located mid-centrally; copulatory ducts originating centrally, sinuous; spermathecae fist-shaped; fertilization ducts transparent. Habitus as shown by Wang et al. (1996).

Male unknown.

##### Distribution.

China (Hunan) (Fig. 7).

#### ﻿ Baiyuerius shenzhen sp. nov.

Taxon classificationAnimaliaAraneaeAgelenidae

B29F5B8A-E401-5456-A9FE-C1FF3B39E245

https://zoobank.org/8CC81DC3-4046-4A37-8161-12F8A08B0BEE

**Figure 4.**
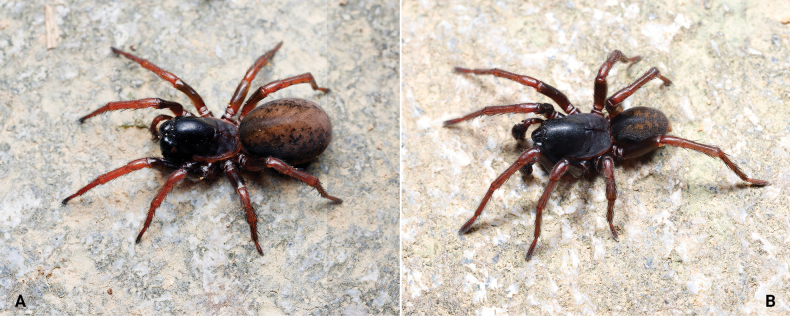
Photo of living *Baiyueriusshenzhen* sp. nov. **A** female **B** male.

**Figure 5.**
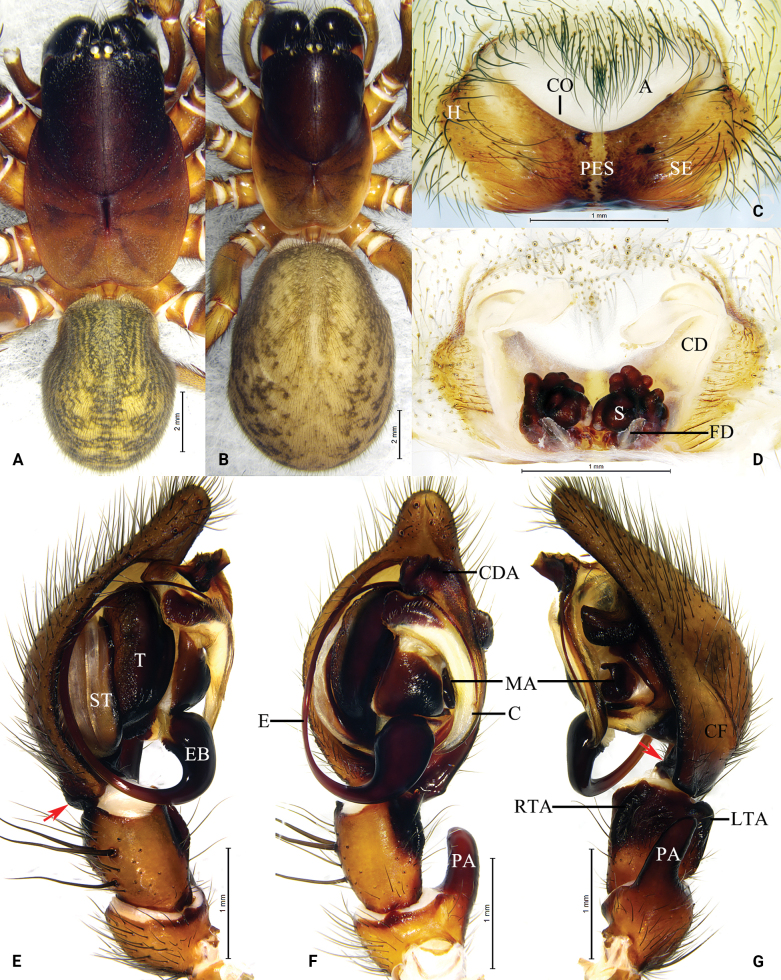
*Baiyueriusshenzhen* sp. nov., male holotype (**A, E–G**) and female paratype (**B–D**) **A** male habitus, dorsal view **B** female habitus, dorsal view **C** epigyne, ventral view **D** same, dorsal view (dash-line indicating the outline of fertilization ducts) **E** left male pedipalp, prolateral view **F** same, ventral view **G** same, retrolateral view. Arrows show the hypophysis of cymbium. Abbreviations: A = atrium; C = conductor; CD = copulatory duct; CDA = dorsal apophysis of conductor; CF = cymbial furrow; CO = copulatory opening; FD = fertilization duc; E = embolus; EB = embolic base; H = hood; LTA = lateral tibial apophysis; MA = median apophysis; PA = patellar apophysis; PES = posterior epigynal sclerite; RTA = retrolateral tibial apophysis; S = spermatheca; SE = swell of epigyne; ST = subtegulum; T = tegulum.

Figs 4 , 5 , 7 (深圳百越蛛)

##### Type materials.

***Holotype*** male (SWUC-T-AG-119-01): China, Guangdong Province, Shenzhen City, Wutong Mountain, Taishanjian, Fenglingjing, 22°34′58″N, 114°11′51″E, elev. 205 m, 16 January 2023, F. Lu leg.; ***Paratype***: 1 female (SWUC-T-AG-119-02), same data as holotype.

##### Etymology.

The specific name refers to the type locality; used as a noun in apposition.

##### Diagnosis.

Male resembles *B.zuojiang* Zhao, Li & Li, 2023 in having a similar, slightly curved patellar apophysis with blunt end, retrolateral tibial apophysis originating at mid-length of tibia, cymbial furrow more than half length of the cymbium (Fig. 4; Zhao et al. 2023: fig. 6A–C), but of *Baiyueriusshenzhen* sp. nov. can be distinguished from the latter by the following: conductor with a smooth surface and flat margin (Fig. 5E–G) vs with jagged margin in *B.zuojiang* (Zhao et al. 2023: fig. 6A–C); cymbial base with two hypophyses (Fig. 5E–G) vs with one hypophysis in *B.zuojiang* (Zhao et al. 2023: fig. 6A–C); patellar apophysis extending above the 2/3 length of tibia (Fig. 5E–G) vs less than 1/2 length of tibia in *B.zuojiang* (Zhao et al. 2023: fig. 6A–C). Female of *Baiyueriusshenzhen* sp. nov. resembles *B.zuojiang* Zhao, Li & Li, 2023 in having a similar atrium, occupying 1/3 of the epigyne, and in the absence of epigynal teeth (Fig. 5D; Zhao et al. 2023: fig. 7A, B), but it can be distinguished from the latter by the following: copulatory opening mid-ventrally in the atrium in the new species (Fig. 5C) vs present in the anterio-lateral margin of the atrium in *B.zuojiang* (Zhao et al. 2023: fig. 7A); spermathecae highly convoluted in new species (Fig. 5D) vs round, fist-like in *B.zuojiang* (Zhao et al. 2023: fig. 7B).

**Figure 6.**
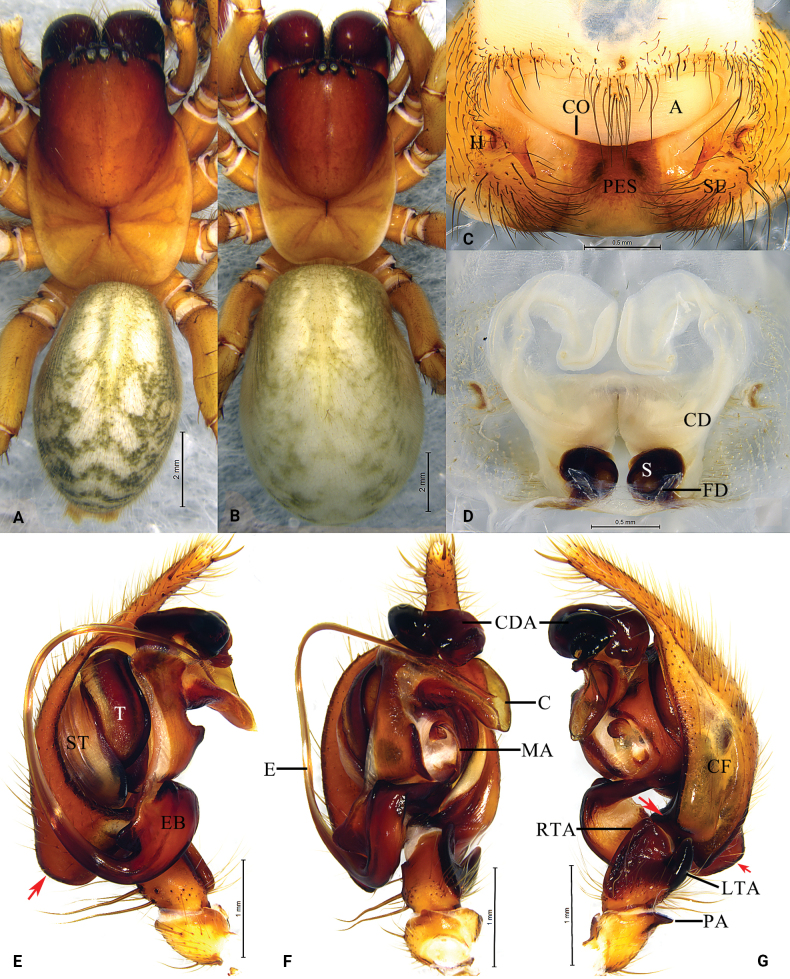
*Baiyueriusyuelu* sp. nov., male holotype (**A, E–G**) and female paratype (**B–D**) **A** мale habitus, dorsal view **B** female habitus, dorsal view **C** epigyne, ventral view **D** same, dorsal view (dash-line indicating the outline of fertilization ducts) **E** left male pedipalp, prolateral view **F** same, ventral view **G** same, retrolateral view. Arrows show the hypophysis of cymbium. Abbreviations: A = atrium; C = conductor; CD = copulatory duct; CDA = dorsal apophysis of conductor; CF = cymbial furrow; CO = copulatory opening; FD = fertilization duct; E = embolus; EB = embolic base; H = hood; LTA = lateral tibial apophysis; MA = median apophysis; PA = patellar apophysis; PES = posterior epigynal sclerite; RTA = retrolateral tibial apophysis; S = spermatheca; SE = swell of epigyne; ST = subtegulum; T = tegulum.

**Figure 7.**
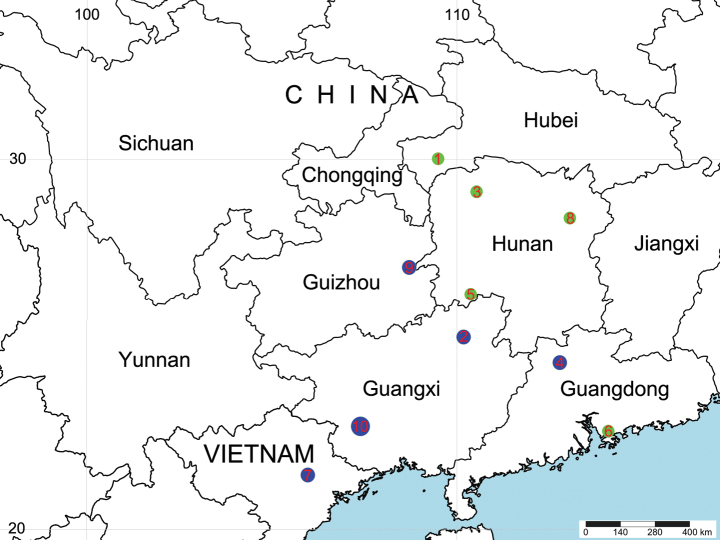
Distribution records of *Baiyuerius* species. 1 = *B.acroprocessus* (Zhang, Zhu & Wang, 2017) comb. nov.; 2 = *B.daxi* Zhao, B. Li & S. Li, 2023; 3 = *B.globasus* (Wang, Peng & Kim, 1996) comb. nov.; 4 = *B.pindong* Zhao, B. Li & S. Li, 2023; 5 = *B.rugosus* (Wang, Peng & Kim, 1996) comb. nov.; 6 = *B.shenzhen* sp. nov.; 7 = *B.tamdao* Zhao, B. Li & S. Li, 2023; 8. *B.yuelu* sp. nov.; 9 = *B.zhuping* Zhao, B. Li & S. Li, 2023; 10 = *B.zuojiang* Zhao, B. Li & S. Li, 2023 in China (blue circles indicate the distribution of species presented by Zhao et al. (2023)).

##### Description.

**Male** holotype (Figs 4B, 5A) total length 14.21. Carapace 8.14 long, 5.46 wide; opisthosoma 5.92 long, 4.47 wide. Carapace brown. Fovea longitudinal. Cervical groove and radial furrows distinct. Eye sizes and interdistances: AME 0.30, ALE 0.31, PME 0.31, PLE 0.33; AME–AME 0.21, AME–ALE 0.25, PME–PME 0.19, PME–PLE 0.45, ALE–PLE 0.11. MOA 0.73 long, front width 0.68, back width 0.77. Clypeus height 0.29. Chelicerae dark, with three promarginal and two retromarginal teeth. Endites and labium black-brown, longer than wide. Sternum brown, with brown hairs, heart-shaped. Legs yellow-brown. Leg measurements: I 22.93 (2.82, 5.64, 2.38, 4.73, 4.51, 2.85); II 20.78 (2.40, 5.06, 2.51, 4.02, 4.17, 2.62); III 17.83 (2.11, 4.34, 2.39, 3.09, 3.75, 2.15); IV 23.28 (2.36, 5.81, 2.38, 4.51, 5.57, 2.65). Leg formula: 4123. Opisthosoma oval. Dorsum yellow-brown, with several black-brown spots. Venter yellow-brown.

Pedipalp (Fig. 5E–G): patellar apophysis finger-shaped with blunt end, extending above more than half the length of tibia; retrolateral tibial apophysis originating from midway along length of tibia; lateral tibial apophysis inconspicuous; cymbial furrow almost half the length of cymbium; cymbial base with two hypophyses; median apophysis semicircular in retrolateral view; embolus dark brown, originating at 6 o’clock; conductor translucent, with a smooth surface; conductor dorsal apophysis dark brown, with serrated margin.

**Female** paratype (Figs 4A, 5B) total length 17.67. Carapace 7.86 long, 5.07 wide; opisthosoma 9.45 long, 6.86 wide. Eye sizes and interdistances: AME 0.27, ALE 0.31, PME 0.30, PLE 0.36; AME–AME 0.15, AME–ALE 0.28, PME–PME 0.11, PME–PLE 0.46, ALE–PLE 0.11. MOA 0.64 long, front width 0.68, back width 0.71. Clypeus height 0.33. Legs yellow-brown. Leg measurements: I 19.36 (2.56, 5.02, 2.36, 4.09, 3.40, 1.93); II 17.54 (2.07, 4.30, 2.28, 3.58, 3.25, 2.06); III 14.29 (1.82, 3.48, 2.01, 2.34, 2.93, 1.71); IV 19.77 (2.16, 5.20, 2.29, 3.79, 4.37, 1.96). Leg formula: 4123.

Epigyne (Fig. 5C, D): atrium glasses-shaped, occupying 1/3 of epigyne; epigynal hood located laterally; swollen area of epigyne rhomboid; copulatory opening located mid-centrally; copulatory ducts originating centrally, extending anteriorly, then curved inward, spermathecae bases close to each other; spermathecae highly convoluted; fertilization ducts transparent, extending laterally.

##### Distribution.

Known only from the type locality, Guangdong, China (Fig. 7).

#### ﻿ Baiyuerius yuelu sp. nov.

Taxon classificationAnimaliaAraneaeAgelenidae

AC814EEE-C3B0-5B9D-9160-22EAC19691A3

https://zoobank.org/AC762E6D-C6C9-42BC-94DF-25659D88ED5C

Figs 6 , 7 (岳麓百越蛛)

##### Type materials.

***Holotype*** male (SWUC-T-AG-118-01): China, Hunan Province, Changsha City, Yuelu Mountain, 28°10′31″N, 112°56′10″E, elev. 83 m, 26 October 2013, L.Y. Wang leg.; ***Paratypes***: 2 males and 1 female (SWUC-T-AG-118-02–04), same data as holotype.

##### Etymology.

The specific name refers to the type locality, used as a noun in apposition.

##### Diagnosis.

Males resemble those of *B.zhuping* Zhao, Li & Li, 2023 in having the similar margin of conductor without any jags, patellar apophysis with a pointed distal end, and cymbial furrow more than half length of the cymbium in pedipalp (Fig. 6; Zhao et al. 2023: fig. 5A–C), but of *B.yuelu* sp. nov. can be distinguished from the latter by the following: conductor dorsal apophysis relatively robust with bifurcated apex and strongly sclerotized (Fig. 6E–G) vs small and light in *B.zhuping* (Zhao et al. 2023: fig. 5A–C); cymbial base with two hypophyses (Fig. 6E–G) vs with one hypophysis in *B.zhuping* (Zhao et al. 2023: fig. 5A–C). Female of *B.yuelu* sp. nov. resembles *B.zuojiang* Zhao, Li & Li, 2023 in having similar, round spermathecae (Fig. 6D; Zhao et al. 2023: fig. 7A, B), but it can be distinguished from the latter by the following: epigyne with conspicuous epigynal teeth in the new species (Fig. 6C) vs epigynal teeth absent in *B.zuojiang* (Fig. 6D; Zhao et al. 2023: fig. 7A); copulatory opening present mid-ventrally in the atrium (Fig. 6C) vs present in the anterio-lateral margin of the atrium in *B.zuojiang* (Zhao et al. 2023: fig. 7A); copulatory ducts strongly curved and with anterior ends almost touching each other (Fig. 6D) vs arc-shaped and with anterior ends laterally distant from each other in *B.zuojiang* (Zhao et al. 2023: fig. 7B).

##### Description.

**Male** holotype (Fig. 6A) total length 12.49. Carapace 6.52 long, 4.44 wide; opisthosoma 6.21 long, 4.00 wide. Carapace yellow-brown. Fovea longitudinal. Cervical groove and radial furrows distinct. Eye sizes and interdistances: AME 0.23, ALE 0.30, PME 0.27, PLE 0.31; AME–AME 0.07, AME–ALE 0.12, PME–PME 0.08, PME–PLE 0.29, ALE–PLE 0.09. MOA 0.70 long, front width 0.51, back width 0.66. Clypeus height 0.17. Chelicerae black-brown, with three promarginal and two retromarginal teeth. Legs yellow-brown. Leg measurements: I 19.39 (2.24, 4.63, 1.84, 4.15, 3.94, 2.59); II 17.62 (1.94, 4.00, 2.00, 3.78, 3.51, 2.39); III 14.49 (1.65, 3.50, 1.59, 2.51, 3.27, 1.97); IV 19.41 (1.88, 4.76, 2.01, 3.88, 4.55, 2.33). Leg formula: 4123. Opisthosoma oval. Dorsum light yellow, with several yellow-brown stripes. Venter brown.

Pedipalp (Fig. 6E–G): patellar apophysis brown and with a pointed distal end; retrolateral tibial apophysis originating from the base of tibia; lateral tibial apophysis finger-like; median apophysis spoon-like; cymbial furrow subequal to 2/3 length of cymbium; cymbial base with two hypophyses; embolus brown, originating at a 6 o’clock position, first quarter widened, then narrowing and wrapped by conductor, embolic base brown, 2 times wider than long; conductor translucent, with a smooth surface and flat margin; conductor dorsal apophysis with bifurcated apex and strongly sclerotized.

**Female** (paratype, Fig. 6B) total length 15.86. Carapace 6.98 long, 4.79 wide; opisthosoma 8.91 long, 6.29 wide. Eye sizes and interdistances: AME 0.19, ALE 0.31, PME 0.28, PLE 0.32; AME–AME 0.10, AME–ALE 0.26, PME–PME 0.10, PME–PLE 0.43, ALE–PLE 0.08. MOA 0.59 long, front width 0.56, back width 0.70. Clypeus height 0.30. Legs yellow-brown. Leg measurements: I 17.38 (2.03, 4.58, 1.92, 3.59, 3.29, 1.97); II 15.98 (1.99, 4.04, 1.82, 3.28, 3.02, 1.83); III 13.73 (1.75, 3.29, 1.88, 2.43, 2.81, 1.57); IV 18.29 (2.17, 4.55, 2.17, 3.54, 3.85, 2.01). Leg formula: 4123.

Epigyne (Fig. 6C, D): atrium glasses-shaped, occupying 1/3 of epigyne; epigynal hood located central-laterally; epigynal teeth distinct, copulatory ducts originating centrally, extending anteriorly, then curved inward, heart-shaped, copulatory opening located mid-centrally; spermathecae coiled to fist-shaped; fertilization ducts transparent, extending laterally.

##### Distribution.

Known only from the type locality, Hunan, China (Fig. 7).

## Supplementary Material

XML Treatment for Baiyuerius acroprocessus

XML Treatment for Baiyuerius globasus

XML Treatment for Baiyuerius rugosus

XML Treatment for Baiyuerius shenzhen

XML Treatment for Baiyuerius yuelu

## ﻿Additional information

### Author ORCIDs

Bin Luo https://orcid.org/0000-0003-0038-8706

Feng Lu https://orcid.org/0000-0001-6291-9117

Zhi-Sheng Zhang https://orcid.org/0000-0002-9304-1789

Lu-Yu Wang https://orcid.org/0000-0002-5250-3473

### Data availability

All of the data that support the findings of this study are available in the main text.
